# Dichlorido[bis­(2-ethyl-5-methyl-1*H*-imidazol-4-yl-κ*N* ^3^)methane]­cobalt(II) monohydrate

**DOI:** 10.1107/S1600536811011032

**Published:** 2011-04-13

**Authors:** Xiao-Min Qian, Yang-Hui Luo, Jin-Feng Li, Shu-Lin Mao, Ge Gao

**Affiliations:** a Ordered Matter Science Research Center, College of Chemistry and Chemical Engineering, Southeast University, Nanjing 210096, People’s Republic of China

## Abstract

In the title compound, [CoCl_2_(C_13_H_20_N_4_)]·H_2_O, the Co^II^ atom lies on a mirror plane and is four-coordinated by two N atoms of the imidazole ligand and two Cl atoms in a distorted tetra­hedral arrangement. The water mol­ecule participates in the formation of hydrogen bonds, resulting in a three dimensional network involving the Cl atoms and the NH groups. The terminal C atom of the ethyl group is disordered over two sites of equal occupancy.

## Related literature

For background to the use of imidazole derivatives as catalyts and biocatalysts for di­oxy­gen transport and electron storage, see: Bouwman *et al.* (2000 ▶). For related structures, see: Beznischenko *et al.* (2007 ▶); Pajunen (1981 ▶). 
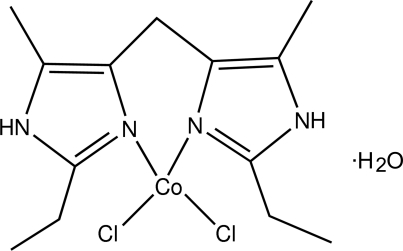


## Experimental

### 

#### Crystal data

[CoCl_2_(C_13_H_20_N_4_)]·H_2_O
*M* *_r_* = 380.18
Monoclinic, 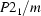

*a* = 8.3927 (7) Å
*b* = 12.1388 (14) Å
*c* = 8.5860 (9) Å
β = 97.045 (1)°
*V* = 868.11 (15) Å^3^
*Z* = 2
Mo *K*α radiation
μ = 1.30 mm^−1^
*T* = 298 K
0.40 × 0.30 × 0.22 mm

#### Data collection

Bruker SMART 1K CCD area-detector diffractometer
Absorption correction: multi-scan (*CrystalClear*; Rigaku, 2005 ▶) *T*_min_ = 0.625, *T*_max_ = 0.763
4313 measured reflections
1607 independent reflections
1174 reflections with *I* > 2.0σ(*I*)
*R*_int_ = 0.039

#### Refinement

*R*[*F* ^2^ > 2σ(*F* ^2^)] = 0.040
*wR*(*F* ^2^) = 0.114
*S* = 1.03
1607 reflections
109 parameters
H-atom parameters constrained
Δρ_max_ = 0.35 e Å^−3^
Δρ_min_ = −0.25 e Å^−3^

### 

Data collection: *CrystalClear* (Rigaku, 2005 ▶); cell refinement: *CrystalClear*; data reduction: *CrystalClear*; program(s) used to solve structure: *SHELXS97* (Sheldrick, 2008 ▶); program(s) used to refine structure: *SHELXL97* (Sheldrick, 2008 ▶); molecular graphics: *ORTEP-3 for Windows* (Farrugia, 1997 ▶) and *PLATON* (Spek, 2009 ▶); software used to prepare material for publication: *SHELXL97*.

## Supplementary Material

Crystal structure: contains datablocks I, global. DOI: 10.1107/S1600536811011032/pv2382sup1.cif

Structure factors: contains datablocks I. DOI: 10.1107/S1600536811011032/pv2382Isup2.hkl

Additional supplementary materials:  crystallographic information; 3D view; checkCIF report

## Figures and Tables

**Table 1:** Hydrogen-bond geometry (Å, °)

*D*—H⋯*A*	*D*—H	H⋯*A*	*D*⋯*A*	*D*—H⋯*A*
O1—H1*C*⋯Cl1	0.85	2.41	3.256 (4)	175
O1—H1*D*⋯Cl2^i^	0.85	2.29	3.139 (4)	175
N2—H2⋯O1^ii^	0.86	2.12	2.959 (3)	165

Symmetry codes: (i) 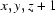; (ii) 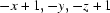.

**Fig. 1.**
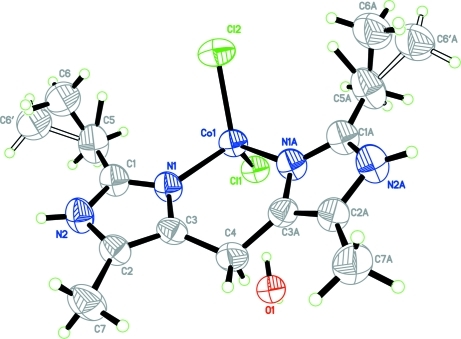
The structure of the title compound with atom-numbering scheme. Displacement ellipsoids are drawn at the 30% probability level. The terminal C-atom of the ethyl group was disordered over sites C6 and C6'. Symmetry code "A" in the labels: x, -y+1/2, z.

## supplementary crystallographic information

### Comment

Imidazole derivatives are used as catalyts and biocatalysts for dioxygen transport and electron storage (Bouwman *et al.*, 2000). As part of our interest in imidazole derivatives, we report here the crystal structure of a new cobalt complex of imidazole derivative.

In the title complex (Fig. 1), the Co^II^ lies on a mirror plane and displays a tetrahedral coordination with two N atoms of the imidazole ligand and two Cl atoms. The asymmetric unit also contains a solvate water molecule. The distances and angles agree with related structures (Beznischenko *et al.*, 2007; Pajunen, 1981). The terminal C-atom of the ethyl group was disordered over two sites C6 and C6' with equal site occopancy factors.

The water molecule participates in the formation of intricated hydrogen bonds resulting in a three dimensionnal network involving the Cl atoms and the NH groups (Table 1).

### Experimental

The ligand and the title complex were prepared by following the procedures reported in the literature (Bouwman, *et al.*, 2000). Single crystals of the title compound as purule prisms were grown from a solution of ethanol by slow evaporation at room temperature within a few days.

### Refinement

Although the atoms  were visible in difference Fourier maps, they were included in the subsequent refinement using restraints. The hydrogen atoms were placed geometrically and treated as riding, with O–H = 0.85 Å, N–H = 0.86 Å, and C–H = 0.96 (methyl) or 0.97 Å (methylene) with *U*_iso_(H) = 1.5*U*_eq_(methyl C) or 1.2*U*_eq_ (the rest of the parent atoms).

### Crystal data

**Table:**

[CoCl_2_(C_13_H_20_N_4_)]·H_2_O	*F*(000) = 394
*M**_r_* = 380.18	*D*_x_ = 1.454 Mg m^−^^3^
Monoclinic, *P*2_1_/*m*	Mo *K*α radiation, λ = 0.71073 Å
Hall symbol: -P 2yb	Cell parameters from 1482 reflections
*a* = 8.3927 (7) Å	θ = 2.4–25.0°
*b* = 12.1388 (14) Å	µ = 1.30 mm^−^^1^
*c* = 8.5860 (9) Å	*T* = 298 K
β = 97.045 (1)°	Prism, violet
*V* = 868.11 (15) Å^3^	0.40 × 0.30 × 0.22 mm
*Z* = 2	

### Data collection

**Table:**

Bruker SMART 1K CCD area-detector diffractometer	1607 independent reflections
Radiation source: fine-focus sealed tube	1174 reflections with *I* > 2.0σ(*I*)
graphite	*R*_int_ = 0.039
Detector resolution: 8.192 pixels mm^-1^	θ_max_ = 25.0°, θ_min_ = 2.4°
φ and ω scans	*h* = −9→9
Absorption correction: multi-scan (*CrystalClear*; Rigaku, 2005)	*k* = −14→13
*T*_min_ = 0.625, *T*_max_ = 0.763	*l* = −5→10
4313 measured reflections	

### Refinement

**Table:**

Refinement on *F*^2^	Primary atom site location: structure-invariant direct methods
Least-squares matrix: full	Secondary atom site location: difference Fourier map
*R*[*F*^2^ > 2σ(*F*^2^)] = 0.040	Hydrogen site location: inferred from neighbouring sites
*wR*(*F*^2^) = 0.114	H-atom parameters constrained
*S* = 1.03	*w* = 1/[σ^2^(*F*_o_^2^) + (0.0528*P*)^2^ + 0.6331*P*] where *P* = (*F*_o_^2^ + 2*F*_c_^2^)/3
1607 reflections	(Δ/σ)_max_ < 0.001
109 parameters	Δρ_max_ = 0.35 e Å^−^^3^
0 restraints	Δρ_min_ = −0.25 e Å^−^^3^

### Special details

**Table:**

Geometry. All esds (except the esd in the dihedral angle between two l.s. planes) are estimated using the full covariance matrix. The cell esds are taken into account individually in the estimation of esds in distances, angles and torsion angles; correlations between esds in cell parameters are only used when they are defined by crystal symmetry. An approximate (isotropic) treatment of cell esds is used for estimating esds involving l.s. planes.
Refinement. Refinement of *F*^2^ against ALL reflections. The weighted *R*-factor *wR* and goodness of fit *S* are based on *F*^2^, conventional *R*-factors *R* are based on *F*, with *F* set to zero for negative *F*^2^. The threshold expression of *F*^2^ > σ(*F*^2^) is used only for calculating *R*-factors(gt) *etc*. and is not relevant to the choice of reflections for refinement. *R*-factors based on *F*^2^ are statistically about twice as large as those based on *F*, and *R*- factors based on ALL data will be even larger.

### Fractional atomic coordinates and isotropic or equivalent isotropic displacement parameters (Å^2^)

**Table:**

	*x*	*y*	*z*	*U*_iso_*/*U*_eq_	Occ. (<1)
Co1	0.75499 (7)	0.2500	0.32643 (8)	0.0443 (3)	
Cl1	0.93574 (14)	0.2500	0.54316 (16)	0.0521 (4)	
Cl2	0.88551 (17)	0.2500	0.11144 (17)	0.0656 (4)	
O1	0.6677 (4)	0.2500	0.7871 (4)	0.0547 (9)	
H1C	0.7331	0.2500	0.7190	0.066*	
H1D	0.7215	0.2500	0.8776	0.066*	
N1	0.5936 (3)	0.1271 (2)	0.3223 (4)	0.0461 (7)	
N2	0.4566 (3)	−0.0235 (3)	0.2690 (4)	0.0525 (8)	
H2	0.4344	−0.0904	0.2413	0.063*	
C1	0.6018 (4)	0.0241 (3)	0.2763 (5)	0.0503 (10)	
C2	0.3496 (4)	0.0527 (3)	0.3131 (4)	0.0446 (9)	
C3	0.4343 (4)	0.1455 (3)	0.3473 (4)	0.0423 (8)	
C4	0.3821 (6)	0.2500	0.4156 (7)	0.0504 (13)	
H4A	0.4209	0.2500	0.5268	0.060*	
H4B	0.2658	0.2500	0.4061	0.060*	
C5	0.7486 (4)	−0.0350 (4)	0.2383 (7)	0.0751 (14)	
H5A	0.8416	0.0099	0.2740	0.090*	0.50
H5B	0.7585	−0.1032	0.2976	0.090*	0.50
H5'A	0.8073	−0.0616	0.3353	0.090*	0.50
H5'B	0.8171	0.0180	0.1941	0.090*	0.50
C6	0.7529 (13)	−0.0595 (9)	0.0839 (15)	0.079 (2)	0.50
H6A	0.6625	−0.1049	0.0469	0.118*	0.50
H6B	0.8504	−0.0982	0.0722	0.118*	0.50
H6C	0.7488	0.0075	0.0240	0.118*	0.50
C6'	0.7231 (13)	−0.1306 (9)	0.1225 (14)	0.079 (2)	0.50
H6'1	0.6592	−0.1062	0.0284	0.118*	0.50
H6'2	0.6688	−0.1895	0.1687	0.118*	0.50
H6'3	0.8252	−0.1562	0.0975	0.118*	0.50
C7	0.1749 (4)	0.0265 (4)	0.3136 (6)	0.0614 (11)	
H7A	0.1211	0.0891	0.3512	0.092*	
H7B	0.1640	−0.0354	0.3809	0.092*	
H7C	0.1279	0.0091	0.2088	0.092*	

### Atomic displacement parameters (Å^2^)

**Table:**

	*U*^11^	*U*^22^	*U*^33^	*U*^12^	*U*^13^	*U*^23^
Co1	0.0267 (4)	0.0526 (5)	0.0533 (5)	0.000	0.0037 (3)	0.000
Cl1	0.0388 (7)	0.0628 (8)	0.0534 (8)	0.000	0.0002 (6)	0.000
Cl2	0.0543 (8)	0.0925 (11)	0.0515 (8)	0.000	0.0126 (7)	0.000
O1	0.051 (2)	0.060 (2)	0.053 (2)	0.000	0.0044 (17)	0.000
N1	0.0271 (14)	0.0473 (18)	0.064 (2)	−0.0015 (12)	0.0038 (13)	−0.0029 (16)
N2	0.0347 (15)	0.0457 (18)	0.076 (2)	−0.0011 (14)	0.0010 (15)	−0.0062 (17)
C1	0.0285 (17)	0.053 (2)	0.068 (3)	0.0012 (16)	0.0006 (16)	−0.003 (2)
C2	0.0331 (17)	0.047 (2)	0.054 (2)	0.0014 (15)	0.0033 (15)	0.0048 (19)
C3	0.0319 (17)	0.046 (2)	0.049 (2)	0.0011 (15)	0.0066 (15)	0.0069 (18)
C4	0.041 (3)	0.052 (3)	0.061 (4)	0.000	0.016 (3)	0.000
C5	0.038 (2)	0.061 (3)	0.126 (4)	0.0039 (19)	0.012 (2)	−0.018 (3)
C6	0.069 (4)	0.083 (7)	0.089 (6)	0.009 (5)	0.030 (4)	−0.008 (6)
C6'	0.069 (4)	0.083 (7)	0.089 (6)	0.009 (5)	0.030 (4)	−0.008 (6)
C7	0.036 (2)	0.062 (3)	0.087 (3)	−0.0042 (18)	0.011 (2)	0.007 (2)

### Geometric parameters (Å, °)

**Table:**

Co1—N1	2.012 (3)	C4—H4B	0.9700
Co1—N1^i^	2.012 (3)	C5—C6	1.363 (13)
Co1—Cl1	2.2520 (14)	C5—C6'	1.526 (12)
Co1—Cl2	2.2590 (16)	C5—H5A	0.9700
O1—H1C	0.8500	C5—H5B	0.9700
O1—H1D	0.8500	C5—H5'A	0.9700
N1—C1	1.316 (5)	C5—H5'B	0.9700
N1—C3	1.398 (4)	C6—H5'B	1.3943
N2—C1	1.343 (4)	C6—H6A	0.9600
N2—C2	1.374 (4)	C6—H6B	0.9600
N2—H2	0.8600	C6—H6C	0.9600
C1—C5	1.496 (5)	C6'—H6'1	0.9600
C2—C3	1.345 (5)	C6'—H6'2	0.9600
C2—C7	1.500 (5)	C6'—H6'3	0.9600
C3—C4	1.486 (4)	C7—H7A	0.9600
C4—C3^i^	1.486 (4)	C7—H7B	0.9600
C4—H4A	0.9700	C7—H7C	0.9600
			
N1—Co1—N1^i^	95.69 (16)	C6—C5—C1	116.0 (6)
N1—Co1—Cl1	113.48 (9)	C1—C5—C6'	117.0 (5)
N1^i^—Co1—Cl1	113.48 (9)	C6—C5—H5A	108.3
N1—Co1—Cl2	112.23 (9)	C1—C5—H5A	108.3
N1^i^—Co1—Cl2	112.23 (9)	C6—C5—H5B	108.3
Cl1—Co1—Cl2	109.28 (5)	C1—C5—H5B	108.3
H1C—O1—H1D	108.3	H5A—C5—H5B	107.4
C1—N1—C3	106.5 (3)	C1—C5—H5'A	108.5
C1—N1—Co1	130.5 (2)	C6'—C5—H5'A	108.8
C3—N1—Co1	122.3 (2)	C1—C5—H5'B	108.1
C1—N2—C2	108.5 (3)	C6'—C5—H5'B	107.0
C1—N2—H2	125.7	H5'A—C5—H5'B	107.1
C2—N2—H2	125.7	C5—C6—H6A	109.5
N1—C1—N2	110.0 (3)	C5—C6—H6B	109.5
N1—C1—C5	126.6 (3)	C5—C6—H6C	109.5
N2—C1—C5	123.4 (3)	C5—C6'—H6'1	109.5
C3—C2—N2	106.1 (3)	C5—C6'—H6'2	109.5
C3—C2—C7	131.8 (3)	H6'1—C6'—H6'2	109.5
N2—C2—C7	122.0 (3)	C5—C6'—H6'3	109.5
C2—C3—N1	108.8 (3)	H6'1—C6'—H6'3	109.5
C2—C3—C4	128.9 (3)	H6'2—C6'—H6'3	109.5
N1—C3—C4	122.0 (3)	C2—C7—H7A	109.5
C3—C4—C3^i^	117.3 (4)	C2—C7—H7B	109.5
C3—C4—H4A	108.0	H7A—C7—H7B	109.5
C3^i^—C4—H4A	108.0	C2—C7—H7C	109.5
C3—C4—H4B	108.0	H7A—C7—H7C	109.5
C3^i^—C4—H4B	108.0	H7B—C7—H7C	109.5
H4A—C4—H4B	107.2		
			
N1^i^—Co1—N1—C1	156.8 (3)	N2—C2—C3—N1	0.8 (4)
Cl1—Co1—N1—C1	−84.6 (4)	C7—C2—C3—N1	−177.8 (4)
Cl2—Co1—N1—C1	39.9 (4)	N2—C2—C3—C4	−173.5 (4)
N1^i^—Co1—N1—C3	−12.2 (4)	C7—C2—C3—C4	8.0 (7)
Cl1—Co1—N1—C3	106.5 (3)	C1—N1—C3—C2	−1.0 (4)
Cl2—Co1—N1—C3	−129.0 (3)	Co1—N1—C3—C2	170.3 (2)
C3—N1—C1—N2	0.7 (4)	C1—N1—C3—C4	173.8 (4)
Co1—N1—C1—N2	−169.5 (2)	Co1—N1—C3—C4	−15.0 (5)
C3—N1—C1—C5	−178.7 (4)	C2—C3—C4—C3^i^	−138.0 (4)
Co1—N1—C1—C5	11.1 (7)	N1—C3—C4—C3^i^	48.3 (7)
C2—N2—C1—N1	−0.3 (5)	N1—C1—C5—C6	−110.0 (7)
C2—N2—C1—C5	179.2 (4)	N2—C1—C5—C6	70.7 (8)
C1—N2—C2—C3	−0.3 (4)	N1—C1—C5—C6'	−153.4 (6)
C1—N2—C2—C7	178.4 (4)	N2—C1—C5—C6'	27.3 (8)

Symmetry codes: (i) *x*, −*y*+1/2, *z*.

### Hydrogen-bond geometry (Å, °)

**Table:**

*D*—H···*A*	*D*—H	H···*A*	*D*···*A*	*D*—H···*A*
O1—H1C···Cl1	0.85	2.41	3.256 (4)	175
O1—H1D···Cl2^ii^	0.85	2.29	3.139 (4)	175
N2—H2···O1^iii^	0.86	2.12	2.959 (3)	165

Symmetry codes: (ii) *x*, *y*, *z*+1; (iii) −*x*+1, −*y*, −*z*+1.
